# Dynamic alterations in cortical activation during motor adaptation in table tennis using whole‑brain fNIRS

**DOI:** 10.1038/s41598-025-94699-3

**Published:** 2025-03-26

**Authors:** Daniel Carius, Elisabeth Kaminski, Martina Clauß, Patrick Ragert

**Affiliations:** 1 Department of Movement Neuroscience, Faculty of Sport Science, Leipzig University, 04109 Leipzig, Germany; 2 Max Planck Institute for Human Cognitive and Brain Sciences, 04103 Leipzig, Germany

**Keywords:** Motor adaptation, Neuroplasticity, Near-infrared spectroscopy, Whole-brain, Table tennis, Sensorimotor processing, Learning and memory, Human behaviour

## Abstract

Human movements must constantly be adapted due to changing internal and external conditions in our environment. The underlying neuronal mechanisms that are responsible for motor adaptations have so far mainly been investigated in highly controlled laboratory scenarios using simple motor tasks. However, because motor adaptations in daily life and sports entail more complex processes involving several cognitive components and strategic adjustments, results from such highly controlled settings only allow restricted conclusions and do not capture neuronal processing in everyday life scenarios. Hence, we studied 56 participants using a table tennis paradigm to unravel cortical activation during motor adaptation in a sport-specific setting using functional NIRS. Furthermore, we wanted to investigate whether cortical activation during motor adaptation is influenced by the temporal order of perturbations (serial vs. randomized practice). Our findings revealed brain areas such as the dorsolateral prefrontal cortex and primary sensory cortex, left supplementary motor cortex and left primary motor cortex, as well as right superior parietal cortex and right inferior parietal cortex, exhibited dynamic alterations in their activation as motor adaptation progressed. Specifically, hemodynamic response alterations generally increased during early adaptation and decreased as motor adaptation progressed. On the other hand, no differential changes in cortical brain processing were observed with serial and randomized practice. Based on our findings, we can confirm and extent theoretical models and laboratory evidence of motor adaptation using a sport-specific motor task.

## Introduction

Human movements frequently require ongoing motor adaptations due to a changing environment. These adaptations represent new sensorimotor relationships and form the basis for human motor behavior. In contrast to new movement skills, adapting already learned internal models to changing physical conditions or new environmental circumstances represent fast learning processes. These adaptational processes usually require only minor changes to previously learned movements^1–5^. Underlying neural mechanisms that are responsible for such adaptations have so far mainly been investigated in highly controlled laboratory settings using EEG or fMRI. However, these methodological approaches allow only limited conclusions for everyday scenarios as motor adaptations in everyday life involve more complex, often non stationary movement patterns with various cognitive components and strategic corrections^6,7^. In the present study, we used a naturalistic motor adaptation paradigm^8^ to unravel hemodynamic response alteration, defined as cortical activation, during motor adaptation in a sport-specific table tennis setting.

### Background on motor adaptation

In general, two mechanisms are involved in motor adaptation: explicit learning driven by target error and implicit learning of a forward model driven by prediction error^9^. During the initial phase of adaptation, predominantly explicit learning takes place in order to minimize target errors. This fast process is followed by slow implicit learning, which gradually updates the sensorimotor mapping. When perturbations disappear after a new sensorimotor mapping, transient after-effects occur, which again represent target errors in the direction opposite to the initial sensorimotor conflict. These after-effects are considered evidence for an adapted internal model that initially persists when perturbation ends^10^. There are two classic approaches that make motor adaptations experimentally accessible. In force-field adaptation tasks^1,2,5,11–14^, the execution of the movement is disturbed by an additional or altered external force. In contrast, visuomotor rotation tasks^3,4,9,15–18^ do not disturb the execution of the movement itself, but the subsequent movement-effect relationship. This creates a mismatch between vision and proprioception that requires adaptations such that individuals have to modify their motor output to account for the respective perturbation and resolve the sensorimotor conflict. In real life scenarios, motor adaptations are sparsely investigated. We recently investigated motor adaptation using a table tennis paradigm. Here, a table tennis robot changed its ball spin during the course of adaptation phases. These environmental changes require sensorimotor adaptations to update the movement-effect relationship while movement execution is not directly affected. In summary, we were able to show that our paradigm reflects typical motor adaptations in a sport-specific context^8^. Furthermore, we observed that on a behavioral level, temporal ordering of the perturbations (serial vs. random practice) had no influence on motor adaptation performance. However, temporal ordering could still induce differential brain processing due to differences in cognitive effort or motivation. Lage et al.^19^ suggested that, despite comparable behavioral performances between serial and random practice schedules, mental resources required for each task may differ. This perspective aligns with findings^20^ indicating that constant and random practice schedules elicit distinct EEG patterns. However, whether serial and random perturbations within our paradigm also produce differential neural activity remains an open question.

### Brain processing during motor adaptation

Evidence from both theoretical models and empirical studies concluded that cerebellar, striatal and neocortical structures such as primary motor cortex (M1), premotor cortex (PMC), dorsolateral prefrontal cortex (DLPFC) and supplementary cortex (SMA) as well as parietal cortex show dynamic alterations during visuomotor adaptation^5,17,21–23^. The cortico-striatal circuit particularly contributes during early motor adaptation, whereas the cortico-cerebellar circuit plays a more critical role during later phases^22^. Among these brain structures, the posterior parietal cortex (PPC) seems to be of particular relevance for successful motor adaptation^7,24^. In general, brain processing increases as a response to a sudden exposure to a perturbation (e.g., visuomotor rotation). On the other hand, brain processing decreases as visuomotor adaptation progresses, and increases again when the perturbation is suddenly removed^17^. These neuronal mechanisms of motor adaptations have mainly been observed using functional magnetic resonance imaging^25,26^. However, Yeo et al.^7^ pointed out that motor adaptation studies using fMRI are usually performed in a supine position due to structural limitations. Since posture directly affects sensorimotor integration and adaptation due to altered somatosensory and vestibular input and only a limited amount of movement is possible during fMRI measurements, one might argue that neural correlations of motor adaptation using fMRI do not necessarily generalize to correlates of adaptations in everyday movements^6,7^. In a recent study, fNIRS was used to investigate changes in cortical activation during motor adaptation in a spatial orientation task^7^. The study showed, that fNIRS in general seems to be feasible to unravel changes in brain processing as a consequence of motor adaptation^7^. However, many everyday and sport-specific motor adaptations take place in non-stationary motor conditions, e.g., during walking. These result from Yeo et al. can therefore not necessarily be generalized to everyday life scenarios or a sport-specific context.

A large number of studies have shown that fNIRS is in fact suitable for investigating cortical activation during the execution of controlled everyday life and sport-specific movements while standing and walking^27,28^. In the present study, we used a table tennis paradigm^8^ to unravel cortical activation during motor adaptation in a sport-specific table tennis setting and to differentiate between temporal order of perturbations. We hypothesized that (a) cortical structures, that are important for motor adaptation such as bilateral M1, PMC, SMA, DLPFC and PPC^5,7,17,21–23^ dynamically alter their activation as motor adaptation progresses. In addition, we hypothesized that (b) hemodynamic response alterations increase during early adaptation, decrease as visuomotor adaptation progresses, and increases again when the rotation is suddenly removed^17^. Furthermore, we hypothesized that (c) serial and randomized practice lead to comparable motor adaptation performances^8,19^ with higher cortical activation during serial compared to random practice^19^. According to the elaboration hypothesis, serial practice demands greater working memory load and sequence integration compared to an unforeseen practice schedule during random practice^19^. Thus, increased cortical activation is expected mainly in the prefrontal cortex, posterior parietal cortex and supplementary motor area, since these regions are mainly involved in cognitive control, motor planning, and sequence coordination^22,24,29^.

## Material and methods

### Participants

We recruited a total of 66 participants. Five participants had to be excluded due to missing data, two participants due to pain and three participants due to issues with the robot. Thus, 56 right-handed healthy volunteers, with an average age of 22.30 ± 2.26 years, ranging from 19 to 27 years old (25 female participants) were included in the present study. The study procedure was approved by the local ethics committee of the University of Leipzig (309/17-ek). Every participant gave written, informed consent, and every procedure followed the guidelines set forth in the Declaration of Helsinki. None of the participants had a history of neurological, psychiatric, cardiovascular, or musculoskeletal diseases, nor had they taken centrally acting medications. To evaluate the effects of motor adaptation during serial and random perturbation situations, participants were randomly divided in two groups. In order to rule out any significant differences between groups with regard to potential confounders, the number of hours that each group spent on sports and fine motor training each week were quantified (see Table 1). All volunteers were found to be right-handed based on the Edinburgh Handedness Questionnaire^30^ with a mean handedness score of 81.65 ± 1.90. The cut-off score of ≥ 50 indicated right-handedness, ≤  − 50 showed left-handedness, and ≥  − 50 indicated ambidextrous handedness^31^. Furthermore, we ensured that each participant was a novice table tennis player who had not taken part in regular table tennis training before. However, participants still needed to be able to execute the required movement skill—the backhand stroke—sufficiently well in predefined, simple conditions (without perturbations). To make sure that participants satisfied this need, we employed a pretest with a predetermined target accuracy (see the following section). The amount of time spent on sports (a) and fine motor training (b) per week—such as playing video games, knitting, crocheting, or playing an instrument—was measured using a standardized questionnaire. All participants completed a visual analog scale (VAS) before and after the experiment to rate their level of attention (1 (very distracted)—10 (very attentive), fatigue (1 (sleeping)—10 (very energetic), and discomfort (1 (no discomfort)—10 (strong discomfort)) in order to account for potential psychological confounders.

**Table 1:** Group demographics.

Group	Age (years)	Gender (female/male)	LQ (score)	Sports/week (hours)	Fine-motor training/week (hours)
Serial *n* = 29	22.38 ± 0.43	14/15	82.21 ± 2.45	4.85 ± 0.47	3.22 ± 0.68
Random *n* = 27	22.22 ± 0.43	10/17	81.04 ± 2.99	4.96 ± 0.56	2.63 ± 0.47

*LQ* laterality quotient as assessed with the Edinburgh Handedness Scale [range: −100 (full left-handed) to + 100 (full right-handed)]. Hours of sports per week and hours of fine motor training per week (e.g., playing a musical instrument, knitting, doing handcrafts, playing video games with a keypad or joystick) were assessed with a questionnaire. All values are depicted as standard error (SE) of the mean. Statistical analysis revealed no differences in age (*U* = 403.5*, p* = 0.849), gender (χ^2^ = 0.72, *p* = 0.396), LQ (*U* = 407.5, *p* = 0.798), sports/week (*U* = 401.0, *p* = 0.882), or fine-motor training/week (*U* = 401.0, *p* = 0.881) between groups.

### Experimental procedure

**Fig. 1:**
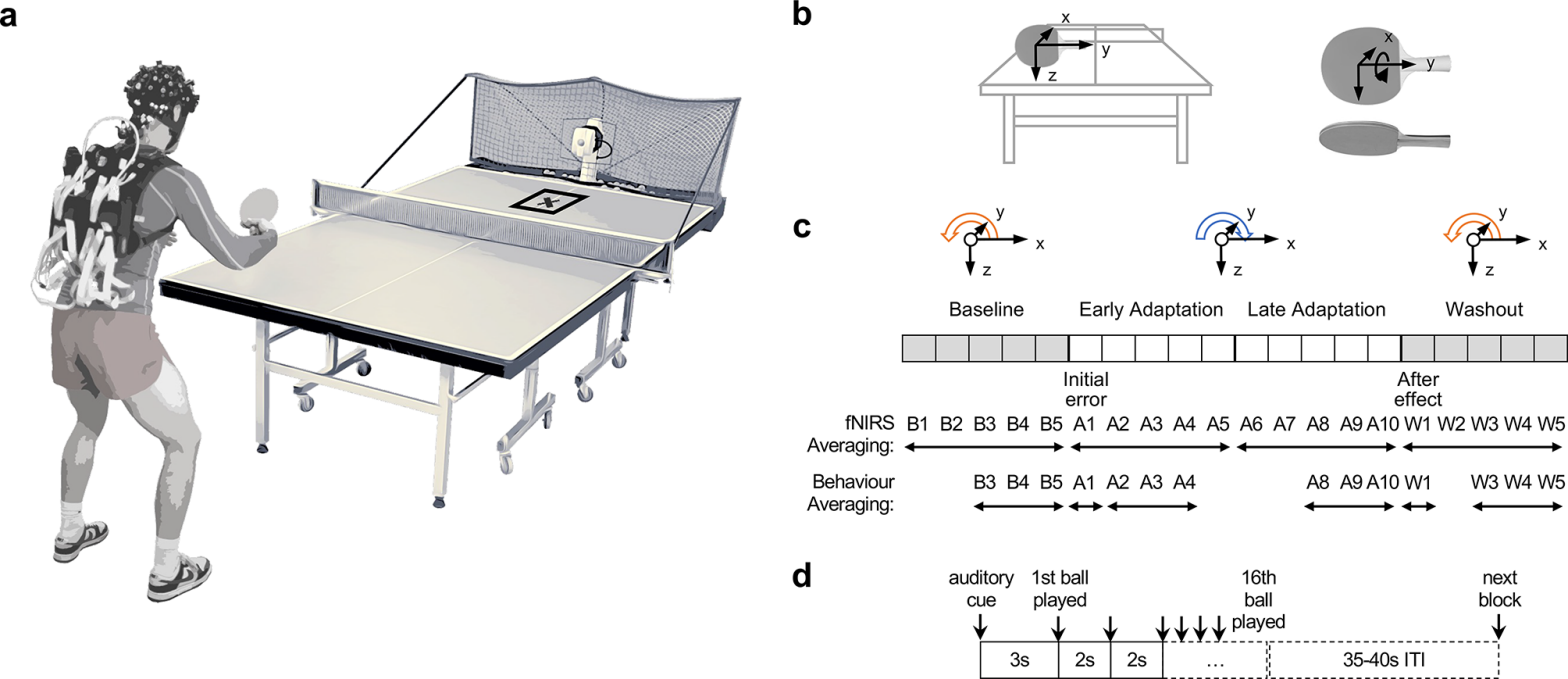
Experimental setup and protocol. (**a**) Positioning of app-controlled ball robot at table tennis plate. Target marked with a cross. Participant in backhand stroke (BH) position carrying the fNIRS system. Photo editing was done using Adobe Photoshop (2024, v. 25) (**b**) Reference system of the racket. Translational movement in x-direction (left panel). Motor adaptation around transversal y-axis (right panel). (**c**) Experimental flow. Ball rotation changes (around transversal y-axis) in the course of adaptation phases. During baseline blocks participants performed BH against topspin balls (orange arrow). During adaptation blocks, participants performed BH against backspin balls (blue arrow), representing disturbances in terms of motor adaptation. In the last five blocks (washout) BH was performed against top spin balls, again. Averaging windows for the behavioral and fNIRS analysis are shown below the block diagram. (**d**) Each block consists of 16 trials (32 s). Blocks were separated by 35–40 s jittered intertrial intervals (ITI).

In the present study, we used a table tennis paradigm^8^ to unravel cortical activation during motor adaptation in a sport-specific setting (Fig. 1a). Participants performed backhand strokes (BH) cross-court against balls played by an app-controlled table tennis robot (Donic Newgy Robo-Pong 3050XL, Germany). During baseline and washout blocks participants performed BH against topspin balls. During adaptation blocks, participants performed BH against backspin balls, representing disturbances in terms of motor adaptation (see Fig. 1b,c). In contrast to our previous study^8^, we standardized the number of task switches for both conditions to improve the comparability of the two protocols. In the current study, we induced eight task switches in both random order and serial order condition (As-As-Al-Al-As-As, As: short ball, Al: long ball) with 16 balls per block being played. BH strokes were executed according to a block design for 20 × 32 s (jittered intertrial interval 35–40 s, 16 balls per block, see Fig. 1c,d), whereby participants were instructed to perform strokes as accurately as possible. Onsets of blocks were indicated via an auditory stimulus (short tone) and fNIRS triggers were set via Psychopy^32^. Motor adaptation was evaluated by means of target accuracy. 2D cinematography was used to determine the placement of participants’ balls. Target accuracy alterations due to adaptation were assessed and distances to the target were computed. Analog to our previously established procedure^8^, target accuracy was recorded with a high-speed video camera (GoPro Hero 9, San Mateo, US, 1920 × 1080 pixel, 200 frames per second,) and ball placement on the table tennis table was measured offline using Kinovea (v0.9.5, GPL-2.0 license).

### Functional near-infrared spectroscopy (fNIRS)

**Fig. 2:**
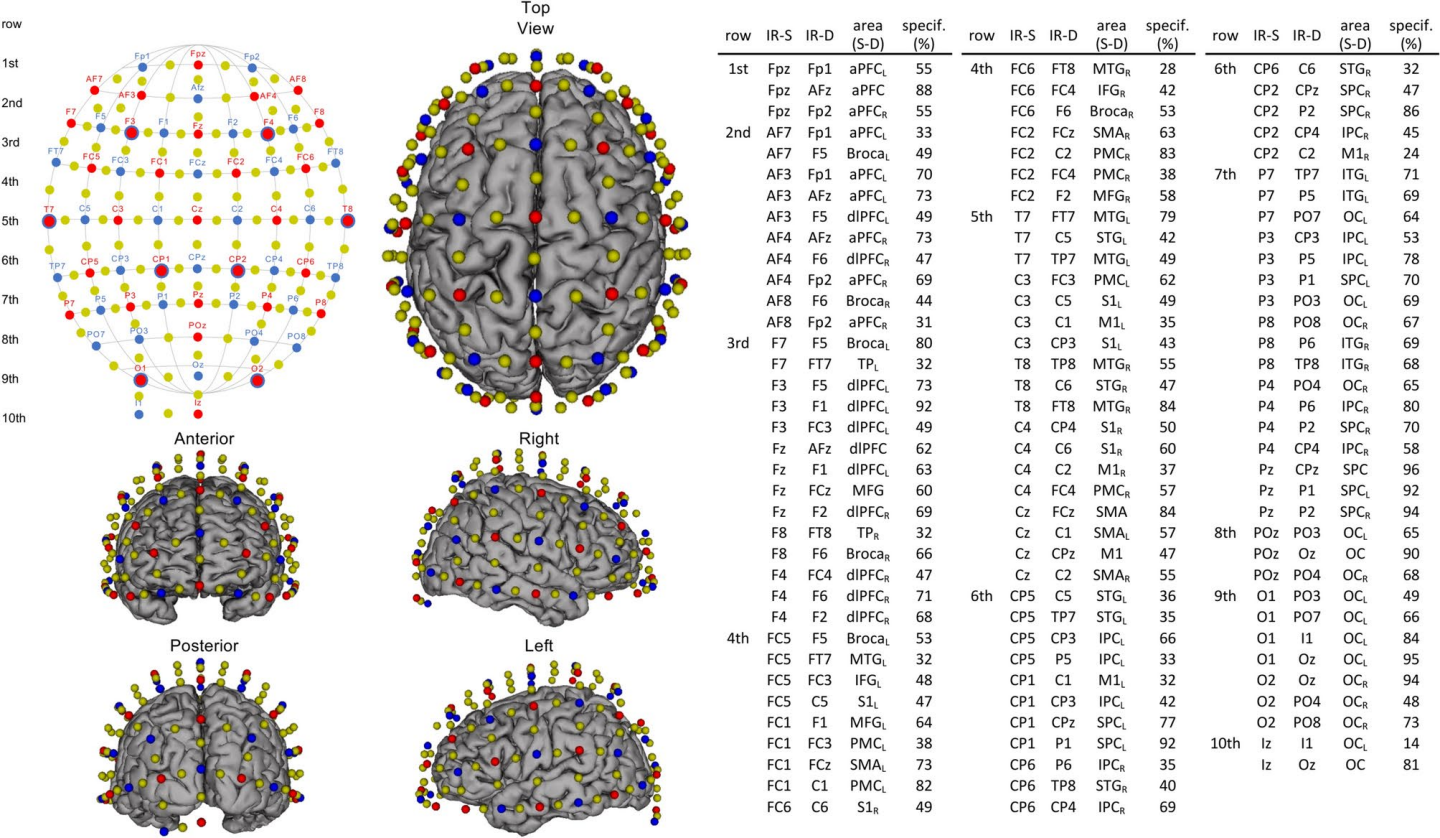
fNIRS configuration used during motor adaptation. Transmitters are shown as red dots and detectors as blue dots. Yellow dots represent each center of the 106 channels (inter-optode distance 35.8 ± 3.9 mm). Short-distance detectors are shown as blue circles surrounding the corresponding transmitters (8 mm distance from F3, F4, T7, T8, CP1, CP2, O1, O2). 10–20 positions for infrared sources (IR-S) and detectors (IR-D). Brain image was created with brain function mapping tool^34^. Respective brain regions, targeted by a 10–20 system transfer method^35^ and defined by the “Brodmann” Atlas (*aPFC* anterior prefrontal cortex, *Broca* broca area, *dlPFC* dorsolateral prefrontal cortex, *IFG* inferior frontal gyrus, *IPC* inferior parietal cortex, *ITG* inferior temporal gyrus, *M1* primary motor cortex, *MFG* middle frontal gyrus, *MTG* middle temporal gyrus, *OC* occipital cortex, *PMC* premotor cortex, *S1* primary somatosensory cortex, *SMA* supplementary motor cortex, *SPC* superior parietal cortex, *STG* superior temporal gyrus, *TP* temporal pole; *L* left hemisphere, *R* right hemisphere).

Hemodynamic responses were recorded using a whole-brain fNIRS system (NIRSport2, NIRX, US). Measurement requirements and fNIRS setup with 32 LED light sources and 32 avalanche photodiode detectors were used analog to Carius et al.^33^. In contrast to our previous study, we used additional washers for avalanche photodiodes (APD). These APD washers (diameter 2.5 cm) are intended to stabilize the APDs and prevent optodes from tilting. To ensure that washers fit under all optodes, we slightly modified the placement of the fNIRS optodes resulting in a more symmetrical configuration (106 actual measurement channels, see Fig. 2). In addition to the 106 (standard) channels, we also used eight short-distance detectors (NIRx Medical Technologies, Glen Head, NY) to exclude potential fNIRS confounders, such as changes in extracerebral blood flow. Data were acquired with a sampling frequency of 10.1725 Hz. To rule out expertise-related differences, we measured cardiac stress, operationalized via heart rate, during the execution of table tennis strokes using a Polar M430 sport watch (Polar Electro Oy, Kempele, Finland) with a sampling frequency of 1 Hz.

### Data analyses

#### Behavioral data

Pythagorean theorem was used to calculate distances between hit points and target point. Distances and centroids, representing direction of deviations, were calculated for each participant and each block^8^. Data analysis and statistical analyses were performed using MATLAB (Version R2023a, MathWorks, Natick, MA, United States of America), RStudio (Version 2023.9.0.463, RStudio Team 2023) and JASP (Version 0.17, JASP Team 2021). Mean distances of blocks B3 to B5 (baseline), A2 to A4 (early adaptation), A8 to A10 (late adaptation), and W3 to W5 (washout) were averaged for statistical examination of adaptation-related performance changes. Mean distances of A1 indicate initial error at the beginning of the perturbation, while mean distances of W1 indicate after-effect. Direction of changes in target accuracy during baseline, perturbation, adaptation, after-effects, and washout are represented by density plots^8^. A 6 (time) × 2 (group) mixed ANOVA with post-hoc testing (i.e., T-tests) was used to examine target accuracy during 1) baseline, 2) initial error, 3) early adaptation, 4) late adaptation, 5) after-effect and 6) washout (see Fig. 1c) for the perturbation groups (serial vs. random). Data were spherically corrected, if needed, using the Greenhouse–Geisser adjustment. The problem of multiple comparisons was mitigated by the application of Holm adjustment.

#### Hemodynamics

FNIRS data analysis was performed in MATLAB using HOMER3 (version 1.80.2)^36^ and QT-NIRS^37^. We used QT-NIRS^37^ to estimate the fNIRS data quality. According to our previously established procedure^33^ (F_minmax_ = [0.5 2.5]; wLength = 3; sciThld = 0.7; pspThld = 0.1), 18 participants had to be excluded. The remaining participants (*n* = 38) showed good data quality for 81.8% of the channels. As a next step, the raw intensity signals were converted to changes in optical density^36^. Wavelet filtering was used to correct for motion artifacts^38,39^. We employed the HOMER3 hmrR_MotionCorrectWavelet filtering function (inter-quartile range 1.22^40^) and the approach outlined by Molavi and Dumont^41^. The data was slightly low-pass filtered with a low pass cutoff frequency of 3 Hz after motion-artifact correction. Since the following GLM tackles this with a polynomial drift correction, we did not employ a high pass filter. Using the modified Beer-Lambert technique (partial pathlength factor: 6.0^36^), attenuation variations of both wavelengths (850 nm and 760 nm) were converted to concentration changes of oxy- and deoxygenated hemoglobin (HbO and HbR, respectively). As advised in the current literature^42^, we report both HbO and HbR (rather than just one chromophore changes). However, HbO and HbR reflect distinct neurophysiological processes related to brain activity. While HbO primarily reflects changes in blood supply, HbR is directly linked to the process of oxygen extraction from the blood^43^. HbO often exhibits larger signal changes and is therefore frequently the main focus of fNIRS studies. However, since HbR concentration changes (i) are less affected by systemic physiological noise^44,45^, (ii) have a stronger correlation with the blood oxygen level-dependent signal of the functional magnetic resonance imaging^46,47^, and (iii) are spatially more focused^44,48^, we primarily, though not solely, focus on the HbR parameter in the results and discussion section.

We used a general linear model approach (GLM) that employs ordinary least squares and a series of Gaussian functions with a standard deviation of 0.5 s and their means separated by 0.5 s over a specific regression time to model the hemodynamic response function (HRF) in order to regress extra-cerebral contaminations (measured by short-distance channels) out of the signal (used parameters in HOMER3 hrmR_GLM function: trange  −2.0 to 40; glmSolveMethod 1; idxBasis 1; paramBasis 0.5 and 0.5). Additionally, we employed a third order polynomial fit to take baseline drift into consideration. The short separation channel, which exhibits the strongest correlation with the corresponding long-separation channel^49,50^, is used for short separation regression (SSR). Time courses of changes in HbO and HbR concentrations in each measurement channel and condition were block-averaged, and single trials were baseline corrected with respect to the two seconds before to stimulus initiation. For TFCE analysis, the complete HbO and HbR time courses were exported.

Statistical Analyses were performed using nonparametric threshold-free cluster enhancement (TFCE, Carius, et al.^33^) with a cluster threshold of p = 0.05^51,52^ and 10.0000 permutations. TFCE tests were conducted in MATLAB using the TFCE-Toolbox^51^. Differences in HbO and HbR concentration changes between motor adaptation phases (baseline vs. early adaptation vs. late adaptation vs. washout) and perturbation groups (serial vs. random) were tested using TFCE mixed two-way ANOVA (default TFCE parameters: E = 2/3, H = 1, Mensen and Khatami^51^, Smith and Nichols^52^). In the case of non-significant interactions, we conducted post hoc tests for the main effects time and group. For the factor time, we used dependent sample TFCE T-Tests (default parameters: E = 2/3, H = 2). For factor group we used independent sample TFCE T-Tests (default parameters: E = 2/3, H = 2).

We report cluster sizes (the number of significant channels and/or time points), corrected p-values and TFCE t-values for the peak coordinates of the resulting clusters. We temporally averaged TFCE t-values^33^ for all channels in the sample range 5 to 32 s in order to illustrate the task-related changes. Finally, we mapped them onto the brain surface using the brain function mapping tool^34^. Spatio-temporal clusters are printed as color-coded T-maps in time and space (106 channels).

## Results

### Behavioral data

**Fig. 3:**
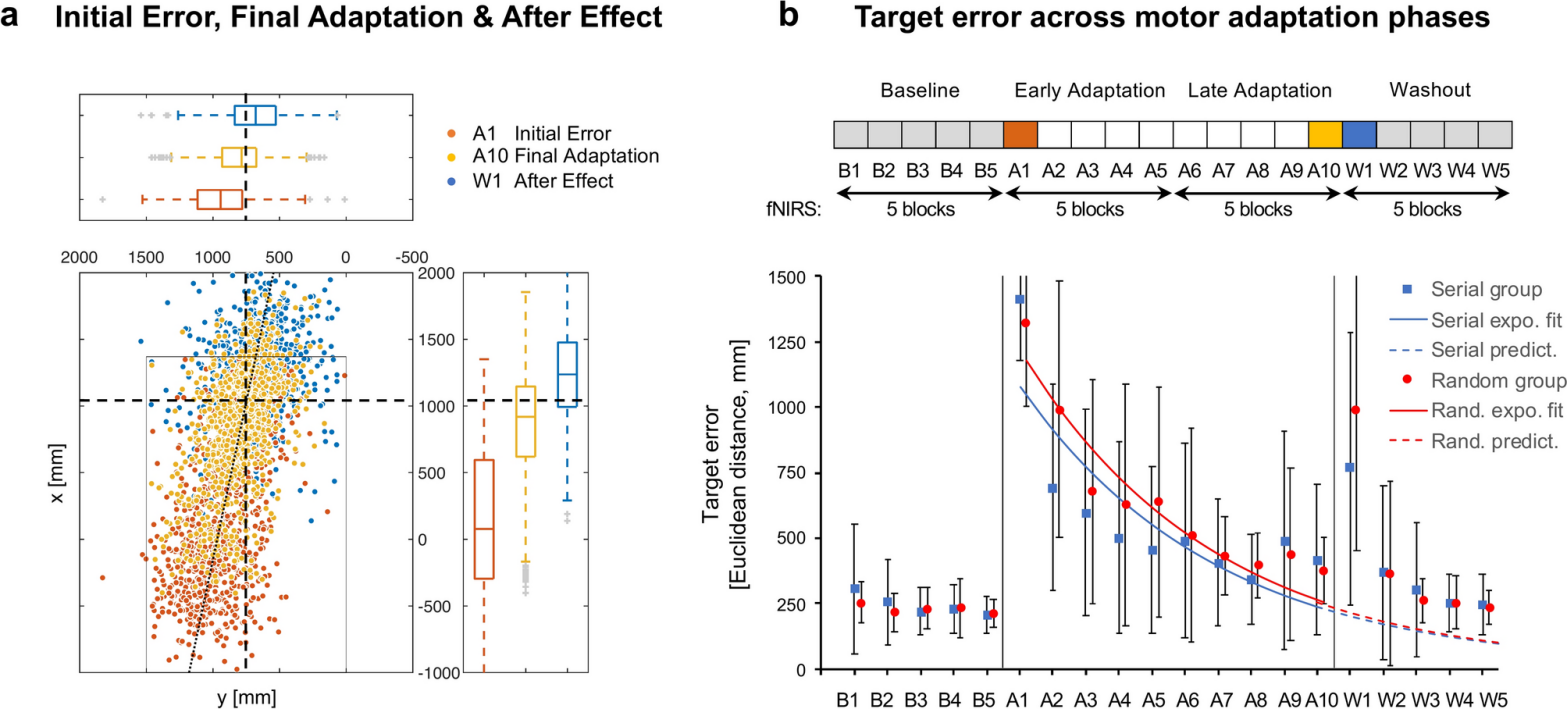
Motor adaptation assessed with table tennis paradigm. (**a**) Dot plot illustrates target error dispersions during initial error (block A1) at perturbation onset, final adaptation (block A10) and after effect (block W1). Display of hit points across 56 participants (896 trials per block: 56 participants × 16 trials). Dottet line represents linear trend. Intersection of dashed lines represents target. Box plots illustrate block-related dispersions in vertical and horizontal direction. Dashed lines show target in vertical and horizontal direction. During perturbation onset majority of balls were played too short resulting in large initial errors (orange). In contrast, when perturbation disappears, majority of balls were played too long resulting in target errors in opposite direction (after effect, blue). Gray box illustrates table tennis table. Hit points outside the table were calculated using data-driven linear model^33^. (**b**) Dot plots and line plots (exponential curve fit: y = a · e^b · x^ ; y = Target error, x = Block; Random group: 139 · e^−0.17 Block^; R^2^ = 0.91; Serial group: 127 · e^−0.17 Block^; R^2^ = 0.66) showing adaptation-dependent performance changes in terms of spatial deviation from target (Euclidean distance) for random and serial group (lower panel). Double arrows below the block diagram illustrate the included data of the phase-related fNIRS block averages (upper panel).

In terms of target accuracy, there was no interaction between time (baseline vs. initial error at perturbation onset vs. early vs. late adaptation vs. after-effects vs. washout) and perturbation (serial vs. random, *F*(2.54, 136.92) = 2.76, *p* = 0.054, *η*_*p*_^*2*^ = 0.05). We also identified no differences between types of perturbation (*F*(1, 54) = 1.06, *p* = 0.307, *η*_*p*_^*2*^ = 0.02). Thus, temporal order of perturbations did not influence motor adaptation. In contrast, we identified adaptation-dependent differences in target accuracy comparing baseline, initial error at perturbation onset, early and late adaptation, after-effects and washout across groups (*F*(2.54, 136.92) = 147.06, *p* < 0.001, *η*_*p*_^*2*^ = 0.73). Post hoc tests revealed an increase in target errors at perturbation onset (baseline (B3-B5)—initial error (A1), *M*_*diff*_ = −114.14, *t*(55) = -22.42, *p*_*holm*_ < 0.001, *d* = −3.85), significant early adaptation (initial error (A1)—early adaptation (A2-A4), *M*_*diff*_ = 68.18, *t*(55) = 13.39, *p*_*holm*_ < 0.001, *d* = 2.30) and late adaptation effects (early adaptation (A2–A4)—late adaptation (A8–A10), *M*_*diff*_ = 27.13, *t*(55) = 5.33, *p*_*holm*_ < 0.001, *d* = 0.91). We observed large target errors when perturbations ends (late adaptation (A8-A10)—after-effect (W1), *M*_*diff*_ = −46.53, *t*(55) = −9.14, *p*_*holm*_ < 0.001, *d* = -1.57) and subsequently significant washout (after-effects (W1)—washout (W3-W5), *M*_*diff*_ = 61.69, *t*(55) = 12.12, *p*_*holm*_ < 0.001, *d* = 2.08). During perturbation onset, the majority of balls were played too short resulting in large target errors. In contrast, when perturbation disappears, majority of balls were played too long resulting in target errors in opposite direction (see Fig. 3a,b).

### Hemodynamics

Nonparametric cluster-based permutation analysis (TFCE) revealed no interactions between motor adaptation phases (baseline vs. early adaptation vs. late adaptation vs. washout) and type of perturbation (serial vs. random), neither for HbO (peak significance found at OC_R_: *F*(1,144)_*max*_ = 5.47, *p*_*max*_ = 0.486, FWE TFCE-corrected), nor for HbR (ITG_R_: *F*(1,144)_*max*_ = 5.07, *p*_*max*_ = 0.726). Additionally, we did not find significant effects for perturbation (HbO, dlPFC_R_: *F*(1,144)_*max*_ = 10.18, *p*_*max*_ = 0.532, HbR, MTG_R_: *F*(1,144)_*max*_ = 8.93, *p*_*max*_ = 0.799). In contrast, cluster-based permutation tests indicated differences between motor adaptation phases (HbO peak significance found at Broca_L_: *F*(3,144)_*max*_ = 17.71, *p*_*max*_ < 0.001, HbR peak significance found at Broca_L_: *F*(3,144)_*max*_ = 14.43, *p*_*max*_ < 0.001).

**Fig. 4:**
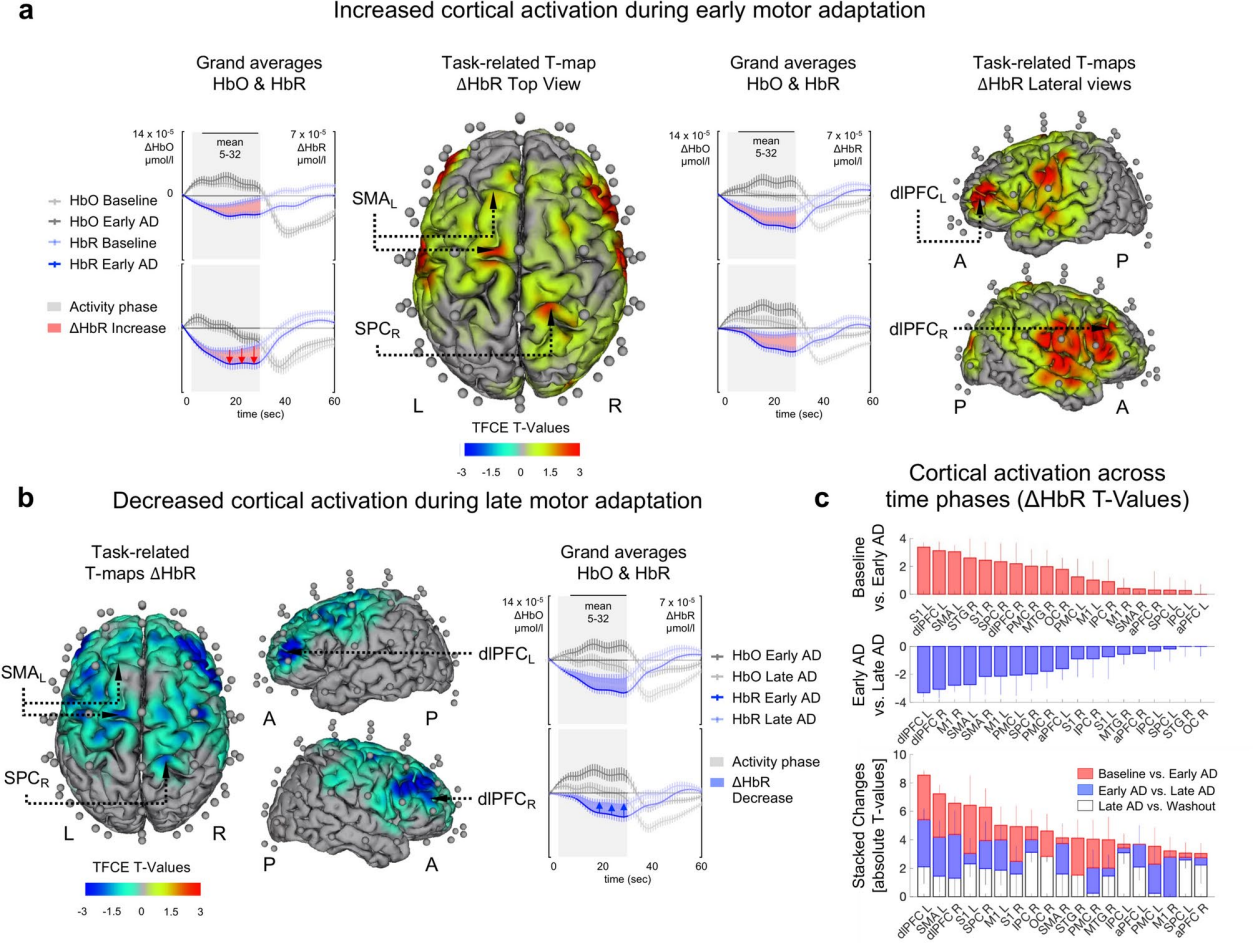
Adaptation-dependent hemodynamic response alterations during table tennis. Line Plots: Grand-averages (HbO & HbR) for ROIs with highest TFCE t-values across time phases. Gray shaded area represents activity phase (0–32 s). T-maps: Temporal aggregated task-related TFCE t-values (HbR). Optodes (transmitters & detectors) are shown for topographic images (created with brain function mapping tool^34^); colors represent mean TFCE t-values (sample range 5–32 s). Images are thresholded at p < 0.05. (**a**) Baseline vs. early adaptation. Red shaded areas between HbR concentration changes as well as topographic difference maps show increased HbR concentration changes during early adaptation compared to baseline indicating higher cortical activation during early adaptation (S1_L_, *t*(37)_max_ = 6.09, *p*_*max*_ < .001). (**b**) Early adaptation vs. late adaptation. Blue shaded area between HbR concentration changes as well as topographic difference maps indicate decreased activation during late adaptation compared to early adaptation (dlPFC_R_, *t*(37)_max_ = 5.20, *p*_*max*_ < .001).(**c**) Cortical activation across time phases (sorted by TFCE t-values). Upper panel: Baseline vs. early adaptation, Centre panel: Early adaptation vs. late adaptation, Lower panel: Summarizing TFCE post hoc comparisons of HbR concentration changes (stacked changes: baseline vs. early AD, early AD vs. late AD, late AD vs. washout), largest overall adaptation-related changes were observed for dlPFC_L_, SMA_L_, dlPFC_R_, S1_L_, SPC_R_, M1_L_, S1_R_, IPC_R_, & OC_R_. *AD* adaptation, *TFCE* threshold-free cluster enhancement, *A* anterior, *P* posterior, *L* left hemisphere, *R* right hemisphere.

TFCE post hoc comparisons of HbR concentration changes indicated higher cortical activation during early adaptation compared to baseline in a widespread cluster (S1_L_, *t*(37)_max_ = 6.09, *p*_*max*_ < 0.001) including S1_L_, dlPFC_L_, SMA_L_, STG_R_, S1_R_, SPC_R_, dlPFC_R_, Broca_R_, PMC_R_, MTG_R_, STG_L_, OC_R_, MTG_L_, PMC_L_, M1_L_, Broca_L_, IPC_R_, M1_R_ & SMA_R_ (76 channels, sorted by temporally aggregated TFCE t-values in descending order, Fig. 4a shows temporal aggregated difference maps for TFCE t-values, Range 5–32 s, and HbO & HbR grand-averages for ROIs with highest t-values). In contrast, the comparison of early and late adaptation indicated lower cortical activation during late adaptation compared to early adaptation in a widespread cluster (dlPFC_R_, *t*(37)_max_ = −5.20, *p*_*max*_ < 0.001) including dlPFC_L_, dlPFC_R_, M1_R_, SMA_L_, SMA_R_, M1_L_, PMC_L_, SPC_R_, PMC_R_, aPFC_L_, S1_R_, IPC_R_, S1_L_, MTG_R_, aPFC_R_, IPC_L_ & SPC_L_ (50 channels, sorted by TFCE t-values in descending order, Fig. 4b shows grand-averages for ROIs as well as temporal aggregated difference maps for all TFCE t-values, Range 5–32 s). Finally, TFCE analysis indicated lower cortical activation during deadaptation/washout compared to late adaptation (dlPFC_R_, *t*(37)_max_ = −4.46, *p*_*max*_ < 0.001, cluster including IPC_R_, IPC_L_, OC_R_, SPC_L_, S1_L_, aPFC_R_, dlPFC_L_, aPFC_L_, SPC_R_, M1_L_, SMA_R_, S1_R_, STG_R_, MTG_R_, SMA_L_, dlPFC_R_, STG_L_, MTG_L_, OC_L_, PMC_R_ & PMC_L_ (73 channels, sorted by TFCE t-values in descending order). Summarizing TFCE post hoc comparisons of HbR concentration changes (TFCE t-values summed across time phases: baseline vs. early adaptation, early vs. late adaptation, late adaptation vs. washout), largest overall adaptation-related changes were observed for dlPFC_L_, SMA_L_, dlPFC_R_, S1_L_, SPC_R_, M1_L_, S1_R_, IPC_R_,& OC_R_ (Fig. 4c).

Regarding HbO concentration changes also indicated higher cortical activation during early adaptation compared to baseline in a widespread cluster (STG_L_, *t*(37)_max_ = 8.29, *p*_*max*_ < 0.001) including STG_L_, S1_R_, S1_L_, STG_R_, MTG_L_, MTG_R_, Broca_R_, ITG_L_, OC_L_, dlPFC_R_, aPFC_R_, Broca_L_, PMC_L_, dlPFC_L_, IPC_L_, M1_R_, OC_R_, ITG_R_, SPC_R_, IPC_R_, M1_L_, PMC_R_, aPFC_L_, SPC_L_ & SMA_R_ (85 channels, sorted by TFCE t-values in descending order). HbO also indicated lower cortical activation during late adaptation compared to early adaptation (aPFC, *t*(37)_max_ = 6.07, *p*_*max*_ < 0.001) including dlPFC_R_, aPFC_R_, PMC_R_, ITG_L_, PMC_L_, Broca_L_, dlPFC_L_, SPC_R_, STG_L_, IPC_L,_ IPC_R_, OC_L_, SPC_L_, Broca_R_, aPFC_L_, OC_R_, M1_R_, STG_R_, SMA_R_, S1_L_, M1_L_, ITG_R_, MTG_R_, MTG_L_ & S1_R_ (102 channels, sorted by TFCE t-values in descending order). Summarizing TFCE post hoc comparisons of HbO concentration changes (TFCE t-values summed across time phases: baseline vs. early adaptation, early vs. late adaptation, late adaptation vs. washout), largest overall adaptation-related changes were observed for STG_R_, MTG_R_, Broca_L_, STG_L_, Broca_R_, S1_R_, dlPFC_R_, S1_L_, aPFC_R_ & ITG_L_.

### Psychological and physiological confounders

Statistical analysis of questionnaires revealed a significant increase for participants’ discomfort (pre: 1.0 ± 0.0 [median ± MAD], post: 2.5 ± 0.5, Wilcoxon signed-rank tests: *z* = −5.28, *p* < 0.001). However, regarding attention (pre: 8.0 ± 1.0, post: 7.0 ± 1.0, *z* = 0.73, *p* = 0.458) and fatigue level (pre: 7.0 ± 1.0, post: 7.0 ± 1.0, *z* =  −1.622, *p* = 0.098), there was no pre-post difference. There were no statistically significant differences between type of perturbation (serial vs. random) concerning pretest (Mann–Whitney U tests: attention: *p* = 0.062, *U* = 283.0, fatigue: *p* = 0.129, *U* = 300.5, discomfort: *p* = 0.804, *U* = 404.5), posttest (attention: *p* = 0.050, *U* = 276.0, fatigue: *p* = 0.052, *U* = 276.0, discomfort: *p* = 0.926, *U* = 385.5), or pre–posttest differences (attention: *p* = 1.00, *U* = 391.5, fatigue: *p* = 0.838, *U* = 379.0, discomfort: *p* = 0.515, *U* = 430.5).

Concerning cardiac stress operationalized via heart rate recordings during the execution of the table tennis strokes, there were no difference between groups (mean ± SD: Serial group: 92.50 ± 12.86 bpm, Random group: 88.32 ± 12.38 bpm; *F*(1, 54) = 1.60, *p* = 0.211, *η*_*p*_^*2*^ = 0.03) and no interaction between time and group (*F*(1.98, 106.82) = 1.40, *p* = 0.250, *η*_*p*_^*2*^ = 0.001). In contrast, the comparison of cardiac stress during baseline, early and late adaptation as well as deadaptation indicated lower heart rates during baseline (baseline: 87.98 ± 12.89 bpm, early adaptation: 91.13 ± 13.04 bpm, late adaptation: 91.25 ± 12.24 bpm; *F*(1.98, 106.82) = 12.72, *p* < 0.001, *η*_*p*_^*2*^ = 0.01, Post Hoc baseline—early adaptation: *t*(55) = −4.68, *p*_*holm*_ < 0.001, d = −0.25, early adaptation—late adaptation: *t*(55) = −0.15, *p*_*holm*_ 1.000, d = −0.01). Even when heart rate is added as a covariate to the model, HbR and HbO response alterations still show a significant increase from baseline to early adaptation (fNIRS subsample, *n* = 38, all channels; HbR: *F*(1, 36) = 5.90, *p* = 0.020, *η*_*p*_^*2*^ = 0.14; HbO: *F*(1, 36) = 29.36, *p* < 0.001, *η*_*p*_^*2*^ = 0.45), indicating an effect independent from heart rate alterations. In addition, covariance analyses were conducted for those channels forming a cluster with significant HbR resp. HbO increases (see previous section). Again, the adaptation dependent increase remains after controlling for the effect of heart rate alterations (HbR: *F*(1, 36) = 9.32, *p* = 0.004, *η*_*p*_^*2*^ = 0.21; HbO: *F*(1, 36) = 41.29, *p* < 0.001, *η*_*p*_^*2*^ = 0.53).

## Discussion

The aim of the present study was to unravel cortical activation during motor adaptation in a sport-specific table tennis setting. While earlier (neuro-)behavioral studies have explored motor adaptation and neural correlates in restricted laboratory settings^21,53^, we are aiming to adress everyday scenarios or sport-specific tasks that involve more complex processes, including various cognitive components and strategic corrections.

In line with our first hypothesis, we observed that (a) cortical structures, in particular, bilateral dlPFC, S1 & PMC, SMA_L_ & M1_L_ as well as SPC_R_ and IPC_R_ dynamically alter their activation as motor adaptation progresses. Specifically, in alignment with theoretical models and laboratory evidence (b) hemodynamic response alterations generally increased during early adaptation and decreases as visuomotor adaptation progresses. Contrary to our assumption, we observed further decreases during washout phase. In accordance with findings of our previous study^8^, we observed typical adaptation-dependent performance changes and the temporal order of perturbations did not influence the process of motor adaptation (hypothesis c). Serial and randomized practice lead to non-differential performance changes and cortical activation.

### ROIs showing adaptation-related response alterations

We observed dynamical changes in cortical activation in bilateral dlPFC, bilateral S1 and bilateral PMC. Furthermore, we identified dynamical changes in SMA_L_ and M1_L_ as well as SPC_R_ and IPC_R_ throughout the adaptation process. The pronounced alterations in bilateral dlPFC and bilateral S1 corroborate the findings of Shadmehr and Holcomb^5,54^. These authors discovered that at the initial stage of the learning process, the capacity of individuals to adapt to a disturbing force field is linked to an elevated activation in the left putamen and bilateral dlPFC. Furthermore, according to the aforementioned study, in the subsequent stages of the adaptation process, the comparison of initial and final learning conditions revealed an activation decline in the left and right sensorimotor cortex and right putamen. We observed an activation increase in bilateral S1 and M1_L_ during early adaptation when compared to baseline and an activation decrease during late adaptation when compared to early adaptation. Moreover, such adaptation-related changes were observed in the SMA_L_. Shadmehr und Holcomb^5,54^ posit, given the significant projections from the sensorimotor cortex to the putamen, that changes observed in these regions are likely related to large-scale reductions in motor output rather than to the acquisition of an internal model. The majority of recent empirical studies have attributed the stepwise encoding of an internal model for novel visuomotor transformations to prefrontal and PPC areas^55^. In this context, our results reinforce the significance of the bilateral dlPFC, where in addition to the initial increase in activation a subsequent decrease in activation was observed.

Furthermore, our investigation of a motor adaptation task in a sport-specific context provides evidence that dynamic alterations occur in PPC throughout the adaptation phase. These findings are specific to the SPC_R_ and the IPC_R_. Generally speaking, the PPC, SPC in particular, facilitates sensorimotor integration by receiving multimodal input from the primary sensory cortex and output from the cerebellum. The IPC is responsible for new sensorimotor transformations and reference maps for spatial orientation and navigation^7^. We observed alterations in the SPC_R_ during early and late adaptation. These findings confirm results from a recent fNIRS study^7^, which suggests that the right posterior parietal cortex (rPPC) may be responsible for the establishment of a visuomotor transformation in the early stages of adaptation. Moreover, lesion studies suggest that rPPC may be specialized for spatial somatosensory function. Moreover, a number of studies have demonstrated the particular importance of the rPPC in the context of visual-spatial attention^56^.

Finally, dPMC is also known to integrate proprioceptive and visual information^17^. When learning new visuomotor associations, both dPMC’s neural activity and cerebellar activation increased^17^ (Doyon et al., 2002). According to Tzvi et al.^17^, the cerebellar dPMC loop has a unique role in controlling the process of visuomotor adaptation. Thus, the dPMC seems to perform visuomotor integration, while the cerebellum most likely represents the internal model of motion. Furthermore, it has been proposed by Hardwick et al. (2015) that dPMC serves as a bridge between visuomotor control and higher cognitive functions during motor learning.

### Direction of the adaptation-related response alterations

Research using fMRI has shown that cerebral activation increases in response to a sudden exposure to a visuomotor rotation, decreases as motor adaptation progresses, and increases again when the rotation is suddenly removed^17,57^. Furthermore, findings using fNIRS indicated that cortical activation initially increased and subsequently decreased after visuomotor adaptation occurs^7,55^. Taken together, empirical evidence suggest, that motor adaptations are linked to enhanced cognitive brain processing. Contrary to our original assumption, we observed decreasing activation during washout phase. This reduced cortical activation may be attributed to the fact that, in contrast to the adaptation phase, after-effects only occur for a brief period and can only be observed to a limited extent with our fNIRS methodology. As evidenced by behavioral data, the occurrence of significant errors is predominantly confined to the initial stage of the washout phase, specifically the initial block (W1). From the second washout-block onward, only minor kinematic errors were observed. However, the observed changes in cortical activation were averaged over five blocks (W1-W5). Therefore, fNIRS block averages do not only reflect a potentially activation increase in W1, but rather an overall enhancement in cortical brain processing across blocks W1-W5.

### Serial vs. randomized practice

In contrast to our previous study, we standardized the number of task switches in the current study. We induced eight task switches in both the random order and the serial order group. Even with this standardized number of switches, we confirm that serial and randomized practice causes comparable contextual interference^58–60^. Also, our observations of cortical brain processing support this finding since serial and randomized practice induce similar changes in brain processing. This finding contradicts our original hypothesis, in which we suspected serial practice to induce higher cortical activation in regions responsible for working memory and sequence integration such as the PFC and SMA. However, according to the action plan reconstruction hypothesis, trial-by-trial skill variations necessitate retrieving information from long-term memory, independent from the factor predictability^61^. Even if participants noticed a predictability in the serial practice schedule, they never the less had to continuously build new motor action plans in each consecutive trial. This is also supported by our behavioral data, which clearly shows comparable contextual interference in both conditions. Furthermore, it might well be that participants didn´t even realized that the serial practice condition included repetitive trials.

The current study is also subject to some constraints. Even though subcortical structures are crucial for motor adaptation, with fNIRS neuroimaging only hemodynamic changes in cortical layers can be detected, making it impossible to determine if motor adaptation also affects subcortical activation (e.g., basal ganglia)^21,25,57,62^. Furthermore, kinematic observations may provide important additional information on the learning process. Particularly changes in hand movement (inclination of the table tennis racket) can be added to the current paradigm relatively easy and may provide important details of the actual movement adaptation. Additionally, the shift from baseline to early motor adaptation phase is not only characterized by a change from topspin (baseline) to backspin (early adaptation) but also by a shift from blocked practice (single ball velocity) to serial or random practice (changing ball velocities). Therefore, contrasting baseline and early adaptation phase in terms of cortical activation does not only portray neural adaptation to the perturbation spin and slightly changing ball trajectories due to the magnus effect but also neural adaptation to the perturbation ball length, induced by changing ball velocities. This was done to increase task complexity to ensure a slow and lasting motor adaptation process that is detectable over a long period of time using our blocked fNIRS protocol. Nevertheless, we observed that these induced complex perturbations led to typical behavioral adaptations, thus would conclude that they also induce typical neural adaptation. Also, the contrast early vs. late adaptation is not affected by this methodological limitation, since in both phases similar complex perturbations were presented. We used a ball robot to standardize the movement task as much as possible. However, this standardization also at least to some extend limits the movement task as compared to match conditions during a real table tennis game. Here, the game situation is constantly changing and the player has to anticipate which shot will be taken next. Technical and tactical information provides clues which action will be performed next (e.g., the opponent’s posture).

Finally, participants reported an increase in discomfort during the time course of the experiment. This in turn might have affected our fNIRS data as well. However, due to the dynamical nature of cortical activation during motor adaptation it seems unlikely that pain-associated brain activation might have contrasted our main findings. Nevertheless, future studies have to be designed to limit the influence of pain-related discomfort and associated brain activations.

## Conclusion

Our whole-head fNIRS study was not only able to demonstrate the involvement of selected regions of interest, e.g., PPC^7^, during motor adaptations. Moreover, it furnished a more comprehensive representation of the continuously evolving cortical activation. In sum, our analysis of cortical brain processing during a complex sport-specific task (in a standing position) corroborates the findings of neurobehavioral studies that observed simple movement tasks under strictly controlled laboratory conditions (in a lying or sitting position).

Our findings revealed that cortical structures, particularly the bilateral dlPFC and S1, the SMA_L_ and M1_L_, as well as the SPC_R_ and IPC_R_, exhibited a dynamic alteration in their activation as motor adaptation progresses. In alignment with theoretical models and laboratory evidence, hemodynamic response alterations generally increased during early adaptation and decreased as visuomotor adaptation progressed. Furthermore, the temporal order of perturbations did not influence the process of motor adaptation. Serial and randomized practice led to the same performance changes. Additionally, no differential changes in cortical brain processing were observed with serial and randomized practice.

## Data Availability

All data that support the findings of this study are available from the corresponding author, D.C. if a formal data sharing agreement exists. Besides, all software used in the present study is open-source and as such publicly available.
